# Comparison of Ultrasonographic, Radiographic, Computed Tomographic, and Ex Vivo Measurements of Feline Femoral Trochlear Groove Depth

**DOI:** 10.3390/ani16182968

**Published:** 2026-09-21

**Authors:** Guilherme Pfeiffer da Silva, Rafael Kretzer Carneiro, Maria Paula Luchi da Silva Mattos, Fábio Trindade Dutra de Almeida Filho, Mariana de Mattos Brose, Marcelo Meller Alievi, Márcio Poletto Ferreira

**Affiliations:** 1 Department of Veterinary Medicine, Federal University of Rio Grande do Sul (UFRGS), Av. Bento Gonçalves, Agronomia, Porto Alegre 91540-000, RS, Brazil; pfeiffervet@gmail.com (G.P.d.S.); fabiofilhoveterinaria@gmail.com (F.T.D.d.A.F.); mariana.radiologia@gmail.com (M.d.M.B.); marcelo.alievi@ufrgs.br (M.M.A.); marcio.ferreira@ufrgs.br (M.P.F.); 2 Laboratory of Diagnostic Imaging, Department of Veterinary, Center for Agroveterinary Sciences (CAV), Medicine, Santa Catarina State University (UDESC), Lages 88520-000, SC, Brazil; maria.mattos@edu.udesc.br

**Keywords:** surgery, stifle, cats, orthopedics, ultrasonography, radiography

## Abstract

Patellar luxation is a common orthopedic condition in cats, and evaluation of trochlear groove depth is important to determine the need for trochleoplasty during surgical planning. This study compared radiography, ultrasonography, and computed tomography with ex vivo measurements, considered the reference method, for the assessment of trochlear groove depth in cats. Twenty-seven stifle joints from 15 healthy adult cats were evaluated. Trochlear groove depth was measured using skyline radiographic projections, ultrasonography with the stifle positioned at 45° and 90° of flexion, computed tomography, and ex vivo evaluation with the stifle flexed at 45° and 90°. Measurements obtained by radiography and ultrasonography with the stifle positioned at 90° did not show significant differences compared with the respective ex vivo measurements at 90°, whereas ultrasonography performed at 45° and computed tomography differed. The results obtained by radiography and ultrasonography at 90° suggest that these techniques provide reliable estimates of trochlear groove depth in healthy cats. These findings support the use of radiography and ultrasonography as reliable imaging methods for preoperative evaluation and surgical planning in cats with patellar luxation.

## Simple Summary

Patellar luxation is a common orthopedic disorder affecting the stifle joint in cats. Preoperative assessment of trochlear groove depth is important for determining whether trochleoplasty is necessary. Reliable imaging techniques can assist in this decision and may help avoid unnecessary trochleoplasty in selected cases. In this study, three imaging modalities (ultrasonography, radiography, and computed tomography) were compared with direct measurements obtained after dissection, which were used as the gold standard. Ultrasonography and radiography performed with the stifle flexed at 90° showed measurements that were more similar to the corresponding ex vivo values obtained at 90°, whereas computed tomography yielded higher measurements.

## 1. Introduction

Patellar luxation is one of the most frequently diagnosed orthopedic disorders in dogs [1,2], whereas in cats, its occurrence is considered less common [3]. When present, clinical signs may include pain, lameness, reduced weight-bearing on the affected limb, decreased jumping ability, and limited extension of the stifle joint [3,4,5]. Patellar luxation can be classified into four distinct grades, with grade 1 representing the mildest form, in which the patella can be manually luxated and returns to the trochlear groove once manipulation ceases, whereas grade 4 represents the most severe form of the condition, in which the patella remains luxated and cannot be manually repositioned into the trochlear groove [2,3].

In domestic cats, medial patellar luxation may be traumatic or developmental in origin [6]. The main abnormalities associated with developmental patellar luxation include trochlear groove flattening, medialization of the tibial tuberosity, reduced proximal femoral anteversion, and other angular limb deformities [6,7]. However, in cats, congenital abnormalities are primarily associated with trochlear groove flattening and hypoplasia of the medial femoral condyle [7,8]. Therefore, assessment of femoral trochlear morphology plays an important role in surgical planning. Femoral trochleoplasty is one of the main procedures used in the surgical treatment of patellar luxation in cats, aiming to deepen the trochlear groove and improve patellar stability [9,10,11,12]. The procedure aims to accommodate 50% of the patellar thickness within the trochlear groove [13]; however, joint exposure may result in damage to the articular cartilage, potentially compromising joint function [14].

Because the decision to perform trochleoplasty depends on an accurate assessment of trochlear morphology, evaluation of the femoral trochlea plays a fundamental role in the surgical planning of patellar luxation. For this purpose, different imaging modalities have been employed, each with specific advantages and limitations. In dogs, computed tomography enables detailed assessment of osseous structures and allows objective measurements of the femoral trochlea, although it has limitations in the evaluation of articular cartilage [15,16]. Additionally, it is an imaging examination associated with higher costs, the need for general anesthesia, and limited availability. In contrast, ultrasonography permits characterization of the articular cartilage, providing a more comprehensive assessment of femoral trochlear morphology. However, ultrasonography is an operator-dependent examination, which should be considered when performing the evaluations [15,17]. Furthermore, recent studies in dogs have demonstrated a positive correlation between trochlear groove depth obtained from tangential radiographic projections and the morphological evaluation of the femoral trochlea [18,19].

Despite these advances, studies comparing different imaging modalities for measuring trochlear groove depth in cats remain limited [7,20], particularly those using ex vivo assessment as the reference standard. Therefore, the aim of the present study was to determine trochlear groove depth in healthy domestic cats using skyline-view radiography, computed tomography at 90° of joint flexion, and ultrasonography at joint flexion angles of 45° and 90°, and to compare these measurements with those obtained from ex vivo evaluation at 45° and 90° of joint flexion. The objective was to provide information that may support preoperative assessment and surgical planning for patellar luxation in felines. The hypothesis of this study was that measurements obtained using the different imaging modalities would be comparable to those obtained through ex vivo evaluation, which was considered the gold standard.

## 2. Materials and Methods

### 2.1. Selection and Description

The study was conducted following approval by the Animal Use Ethics Committee of the Federal University of Rio Grande do Sul (protocol No. 44620). A total of 27 pelvic limbs from 15 adult cats, aged 2 years or older and with a mean body weight of 3.34 ± 0.23 kg, were included. The animals were euthanized for reasons unrelated to the study. Prior to inclusion in the study, all limbs were evaluated through an orthopedic examination. Limbs presenting orthopedic abnormalities, such as patellar luxation, fractures, radio-graphic meniscal lesions (meniscal mineralization) or ligament injuries, were excluded. At the final stage, three limbs from three different cats were excluded due to unilateral meniscal mineralization. All clinical evaluations were performed by a veterinarian specialized in veterinary orthopedics (M.P.F.).

Radiographic assessment was performed using conventional orthogonal projections to select only the stifle joints without evidence of osteoarticular abnormalities, fractures, open growth plates, or significant angular deviations. Following selection, the cadavers were kept frozen and subsequently thawed at room temperature for 24–36 h before the experimental procedures.

### 2.2. Radiographic Evaluation

Radiographic examinations were performed using a digital radiography (DR) system (Siemens^®^, München, German) and evaluated using QXLink image viewing software (Version 3.3).

**Figure 1:**
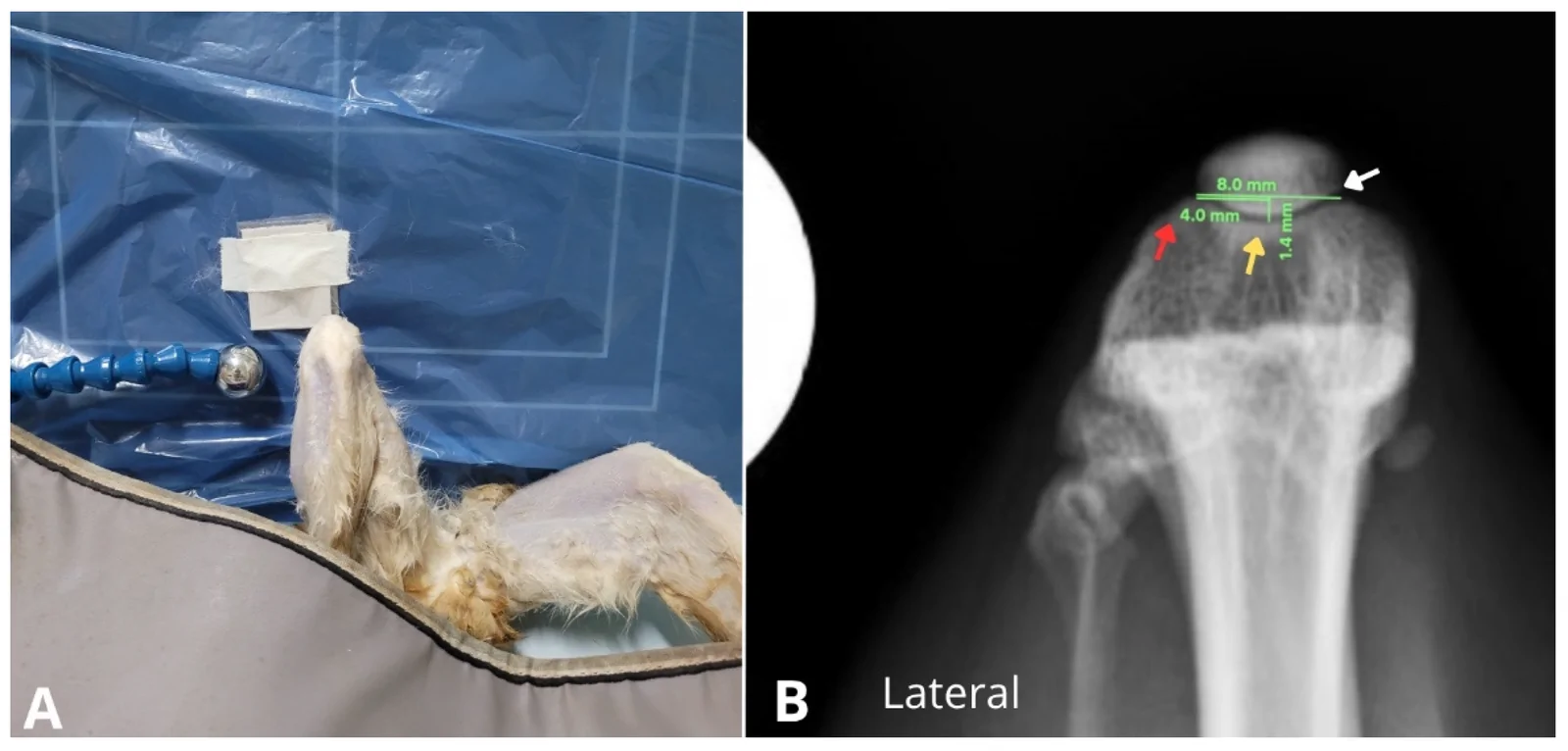
Skyline radiographic projection of the stifle joint (**A**) followed by radiographic assessment in a cat. In (**B**), a line tangent to the femoral trochlear ridges (white arrow) is initially drawn, measuring 8 mm. Next, a line parallel to the first is drawn to determine the midpoint of the trochlear groove (4 mm) (red arrow). Finally, the trochlear groove depth is measured by extending a line perpendicular to the initial line to the base of the trochlear groove (yellow arrow).

A tangential craniocaudal (skyline) radiographic projection was obtained for all limbs. The stifle joint was maintained in maximal flexion, and the radiographic detector was positioned vertically cranial to the femur to allow visualization of the femoral trochlear groove. Radiographic images were obtained using a 2.54 cm circular metallic radiographic marker positioned at the level of the stifle joint. A horizontal X-ray beam was used to obtain the skyline projection images (Figure 1). Radiographic measurements were performed using vPOP Pro software (Veterinary Preoperative Orthopaedics Planning, version 2.9.2, VETSOS Education LTD., Shrewsbury, UK). Image magnification was corrected to obtain the true trochlear groove depth (TGD). TGD was determined by drawing a horizontal line tangential to the femoral condyles and a second line perpendicular to the first, positioned at the midpoint of the femoral trochlea and extending to the base of the trochlear groove. The length of this perpendicular line was recorded as the TGD (Figure 1). All images were evaluated by a single evaluator (R.K.C.).

### 2.3. Ultrasonographic Evaluation

Ultrasonographic examination was performed using an HS30 ultrasound system (Samsung^®^, Seoul, Republic of Korea) equipped with a 10–16 MHz multifrequency linear transducer. The stifles were clipped prior to imaging, and ultrasound gel was applied to the skin to minimize artifact formation. Gain, depth, and focal settings were individually adjusted for each stifle.

Ultrasonographic evaluation was performed with the stifle joint positioned at two different degrees of flexion. Initially, the stifle was flexed at 45°, and the transducer was positioned proximal to the patella to measure trochlear groove depth (TGD). Subsequently, the joint was flexed at 90°, and a second TGD measurement was obtained with the transducer positioned distal to the patella.

**Figure 2:**
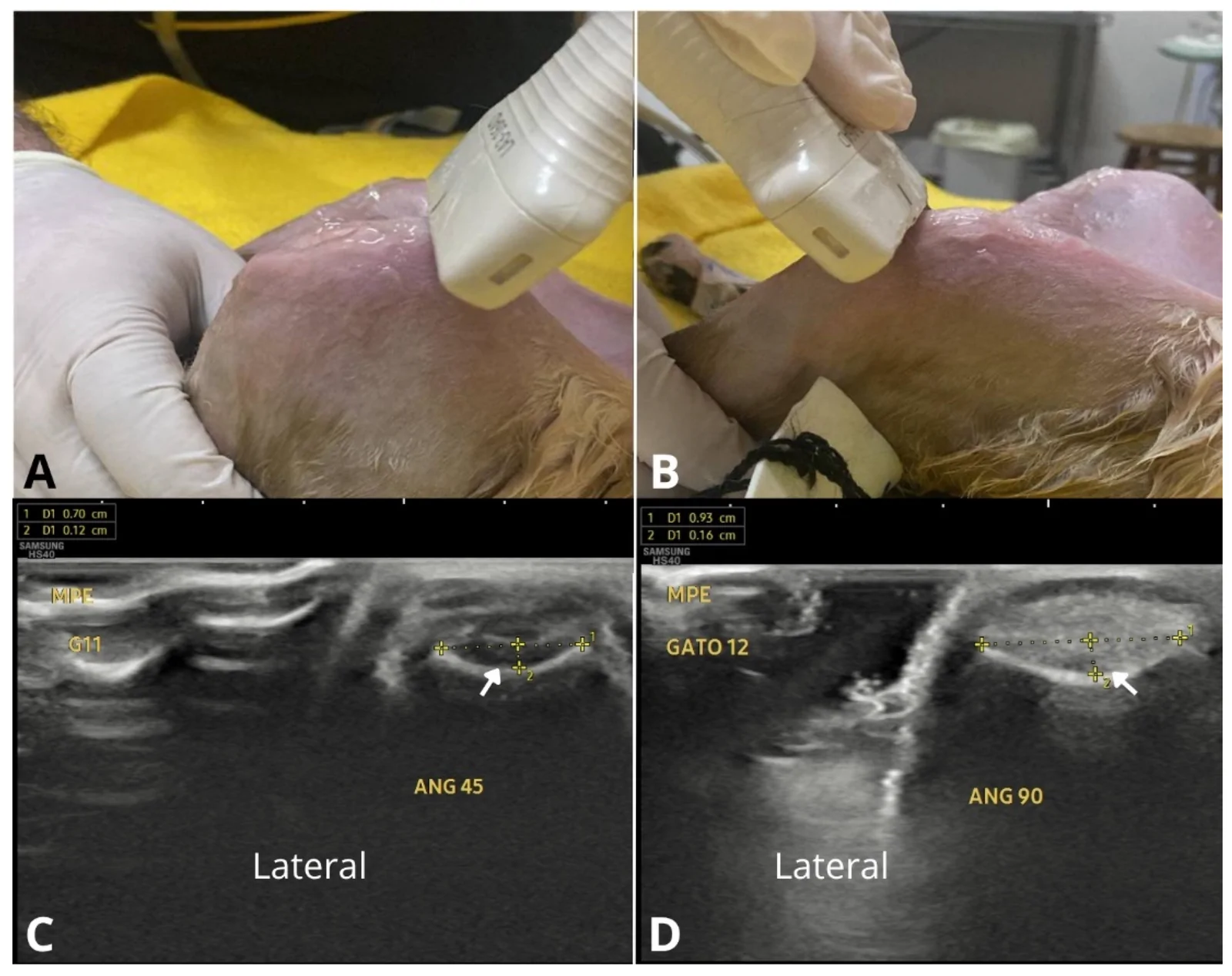
Ultrasonographic evaluation of the feline stifle joint. (**A**) Demonstration of transducer positioning with the stifle joint flexed at 45°, with the transducer placed proximal to the patella. (**B**) Demonstration of transducer positioning with the stifle joint flexed at 90°, with the transducer placed distal to the patella. (**C**) Ultrasonographic measurement of the trochlear groove depth at 45°, demonstrating a value of 0.12 cm (white arrow). (**D**) Ultrasonographic measurement of the trochlear groove depth at 90°, demonstrating a value of 0.16 cm (white arrow).

To ensure proper limb positioning, joint angulation was determined using a 200 mm digital goniometer placed on the lateral aspect of the limb. The stifle joint was used as the central reference point, while the greater trochanter of the femur and the tarsus were considered the proximal and distal reference points, respectively. During all ultrasonographic evaluations, the transducer was oriented transversely to the trochlear groove and maintained perpendicular (90°) to the femoral trochlea (Figure 2). The pressure applied to the transducer was not quantified during the evaluations. Trochlear groove depth (TGD) was determined as the perpendicular distance from the deepest point of the cartilaginous surface of the trochlear groove to a reference line connecting the highest points of the medial and lateral trochlear ridges (Figure 2). The images were acquired and measured by a single evaluator (G.P.S.). The trochlear groove was measured once in each stifle, and the mean of the measurements was subsequently calculated.

### 2.4. Computed Tomographic Evaluation

**Figure 3:**
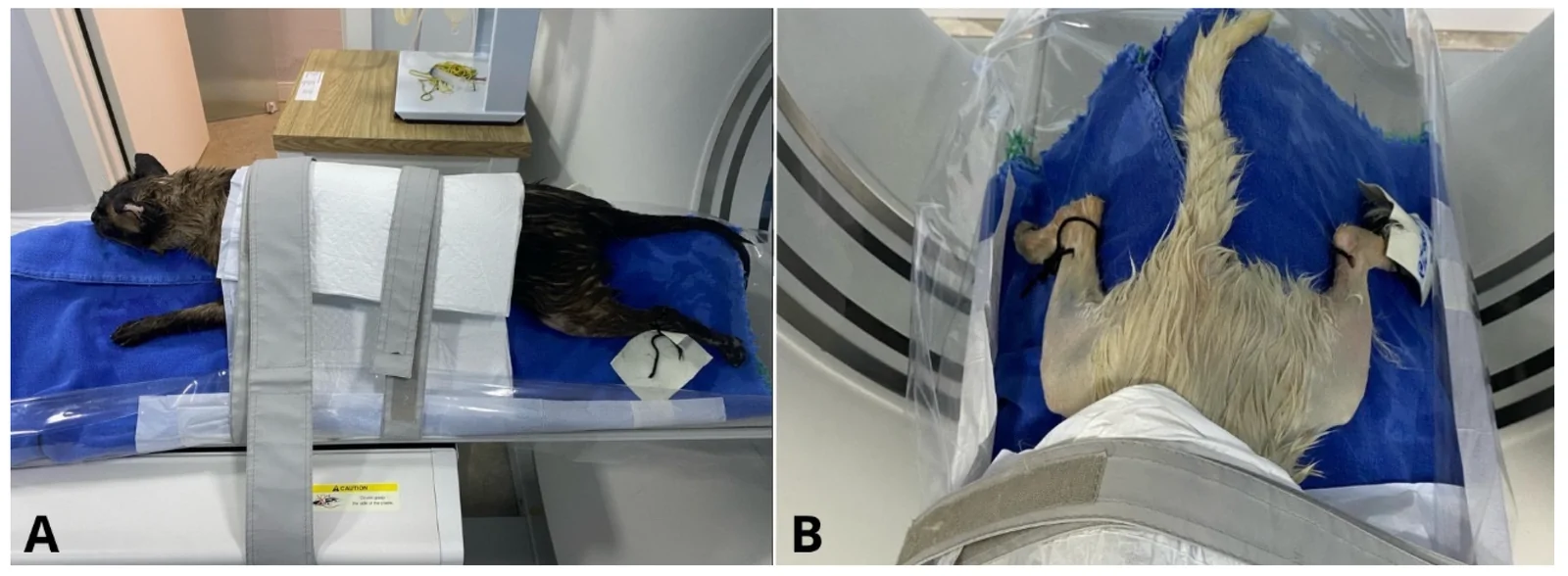
Positioning of a cat for computed tomography of the pelvic limbs with emphasis on the stifle joint. (**A**) Lateral view. (**B**) Dorsal view.

Computed tomography images were acquired using an 8-slice Revolution ACT CT scanner (GE Healthcare®, Chicago, IL, USA). The animals were positioned in sternal recumbency on a foam trough, with the pelvic limbs positioned caudally and the stifle joint flexed at 90° (Figure 3). Volumetric images were acquired with a slice thickness of 1.25 mm, allowing multiplanar and three-dimensional reconstructions. Following image acquisition, DICOM files were analyzed using RadiAnt DICOM Viewer software (version 2023.1). Trochlear groove depth was measured using the multiplanar reconstruction tool, which allowed the acquisition of axial plane images and the identification of trochlear depth. The trochlear groove depth was measured distal to the patellar region. The images were reconstructed using the bone window settings for computed tomography. All images were acquired and evaluated by a single evaluator (R.K.C.) using the same settings described above.

**Figure 4:**
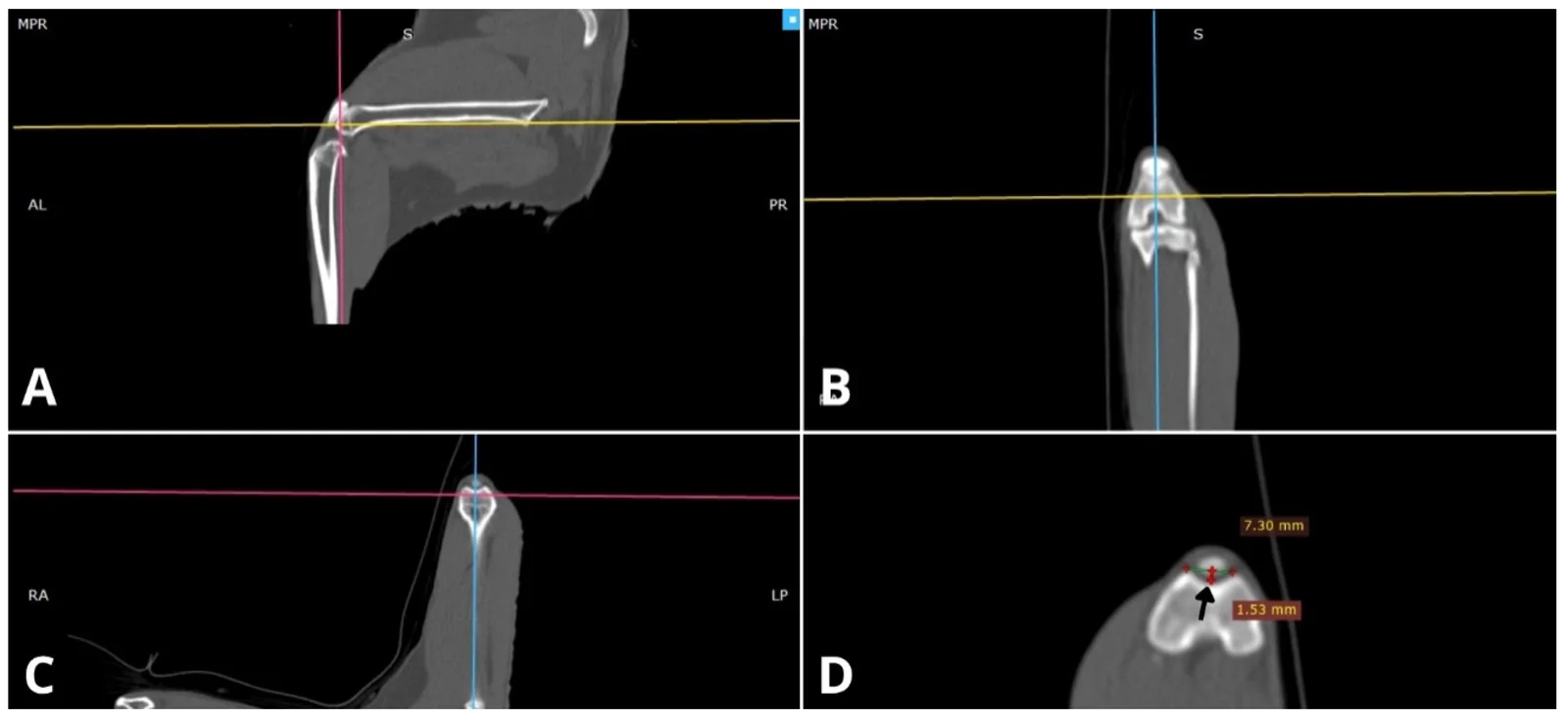
Computed tomographic evaluation of the feline stifle for measurement of the trochlear groove depth. (**A**) Sagittal plane of the femur. (**B**) Coronal plane of the femur. (**C**) Axial plane of the femur. (**D**) Measurement of the trochlear groove depth (black arrow), demonstrating a value of 1.53 mm.

Trochlear groove depth was determined by drawing a horizontal line tangential to the femoral condyles and a second line perpendicular to the first, extending from the central portion of the groove to the base of the femoral trochlea. The length of the perpendicular line was used as the measurement of trochlear groove depth (Figure 4).

### 2.5. Arthrotomy and Measurement with a Caliper

**Figure 5:**
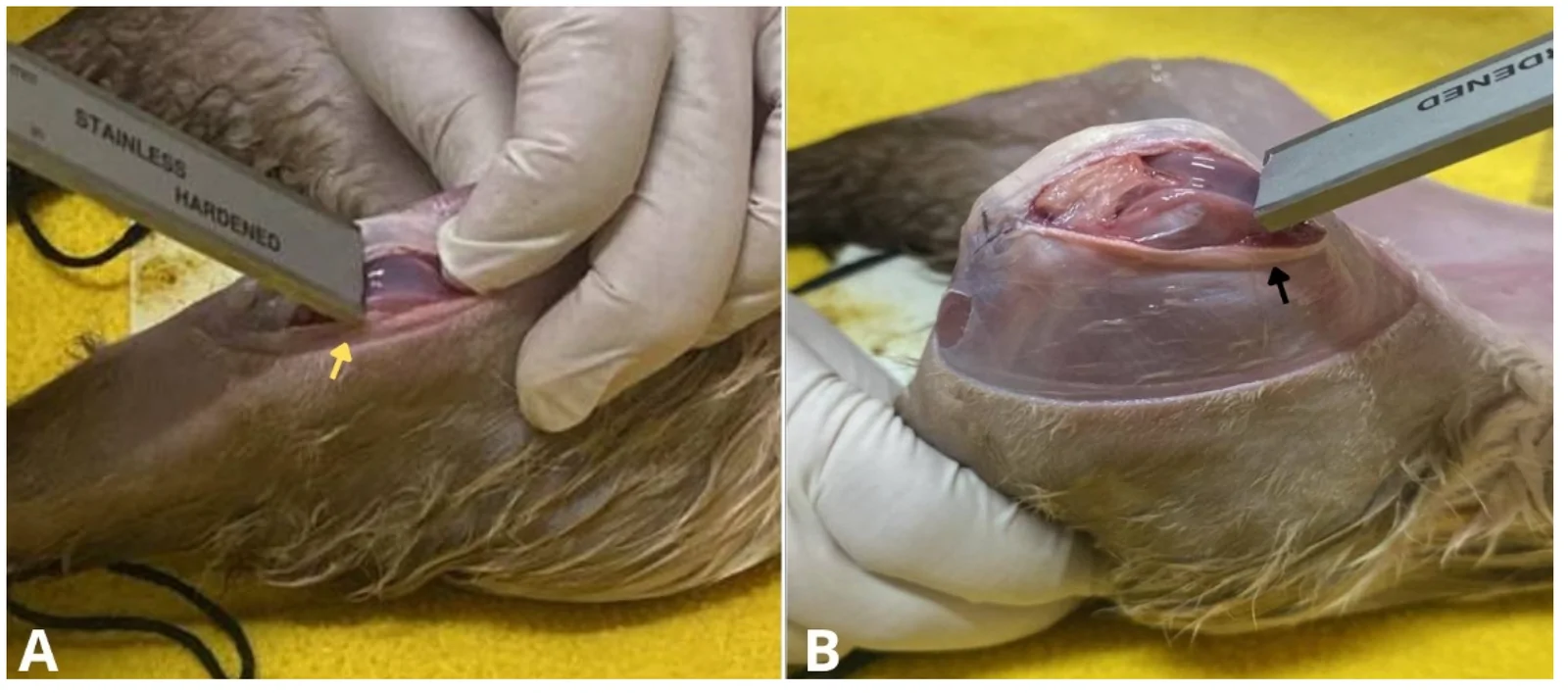
Ex vivo measurement of the trochlear groove depth in a feline stifle using a digital caliper. (**A**) Measurement of the trochlear groove depth with the stifle joint flexed at 90°, with the digital caliper positioned distal to the patella (yellow arrow). (**B**) Measurement of the trochlear groove depth with the stifle joint flexed at 45°, with the digital caliper positioned proximal to the patella (black arrow).

After completion of the imaging assessments, the trochlear groove was exposed through arthrotomy to allow direct in situ measurement. A digital caliper (Davidson^®^, Berlin, Germany; display resolution: 0.01 mm) was used for this purpose. Joint positioning and flexion angles were maintained according to the protocol established for the ultrasonographic examination. The joint was accessed through a longitudinal skin incision located lateral to the stifle, followed by medial luxation of the patella to allow visualization of the articular structures. During measurement, the caliper was maintained perpendicular to the femoral trochlea, reproducing the orientation used during the ultrasonographic assessment (Figure 5).

### 2.6. Data Recording and Statistical Analysis

All images were randomized prior to evaluation, and none of the evaluators had access to the measurements performed by the others. All data were entered into Microsoft Excel and subsequently exported to SPSS version 20.0 for statistical analysis. Categorical variables were described using frequencies and percentages. The normality of quantitative variables was assessed using the Shapiro–Wilk test. Quantitative variables with a normal distribution were expressed as mean ± standard deviation. Measurements were compared between sides and between imaging examinations and macroscopic analysis using the paired Student’s *t*-test. Comparisons among imaging modalities were performed using repeated-measures analysis of variance (ANOVA), followed by Bonferroni post hoc testing. A significance level of *p* < 0.05 was considered for all comparisons.

## 3. Results

Initially, 15 animals were enrolled in the study. However, only 27 pelvic limbs were included in the analysis. Three limbs were excluded due to meniscal mineralization, identified unilaterally in three different cats. Mixed-breed cats predominated, accounting for 85.72% (n = 12) of the study population, followed by one Exotic Shorthair cat (7.14%) and one Siamese cat (7.14%). Females were more prevalent, representing 64.3% of the animals included in the study. The mean body weight was 3.61 ± 0.80 kg. 

Measurements obtained from the macroscopic evaluation of the femoral trochlear groove were considered the gold standard for trochlear groove depth assessment. Therefore, all imaging techniques were compared with the dissected femoral trochlear groove (DFTG). During the comparison of the imaging methods, similarity was observed between the radiographic evaluation and the ex vivo measurement at 90° (*p* = 0.157), as well as between the ultrasonographic evaluation at 90° and the ex vivo measurement at 90° (*p* = 0.832). Comparative data regarding trochlear groove depth obtained from the different imaging modalities and the dissected femoral trochlear groove are presented in Table 1. Comparison of the mean femoral trochlear groove depths between the right and left limbs revealed no statistically significant differences (Table 2). During the comparison between the imaging modalities, similarity was observed between the radiographic and ultrasonographic evaluations at 90° (*p* = 0.667) and between the radiographic evaluation and computed tomography (*p* = 0.999). The imaging modalities compared are presented in Table 3.

## 4. Discussion

This study enabled the evaluation of trochlear groove depth in cats without joint abnormalities using three different imaging techniques, comparing their results with ex vivo evaluation, considered the gold standard. Therefore, the findings provide new perspectives for the assessment of trochlear groove depth in this species. After analysis of the results, the study hypothesis was partially rejected, since similarity with the ex vivo evaluation was observed only for measurements obtained by radiography and ultrasonography with the stifle positioned at 90° of flexion, compared with ex vivo measurements performed at 90°.

Assessment of trochlear groove depth represents an important step in the surgical planning of patellar luxation in cats [3,4,5,6,7,8,9,10,11,12,13,14,15,16,17,18,19,20,21]. This relevance is attributed to the fact that cats with patellar luxation tend to present a relatively shallower trochlear groove compared with healthy cats [7]. Although several studies have described methods for evaluating the trochlear groove in dogs [22,23,24,25], research in felines remains limited [7,8,9,10,11,12,13,14,15,16,17,18,19,20], with a lack of objective parameters applicable to clinical practice and surgical planning in this species.

Trochleoplasty prolongs surgical time and increases patient morbidity because it represents an additional intervention [26]. Furthermore, prolonged surgical procedures may increase the rate of surgical site infections [27]. In dogs, complications associated with trochleoplasty include osteochondral block migration following wedge trochleoplasty and medial or lateral trochlear ridge fractures following block trochleoplasty [28,29]. Quantitative values obtained through imaging examinations may contribute to a more objective assessment of trochlear groove morphology, reducing the subjectivity inherent in visual evaluation. This approach may be particularly relevant in borderline clinical cases in which trochlear groove morphology is equivocal and the decision to perform trochleoplasty may be challenging. Therefore, imaging methods capable of objectively assessing trochlear groove depth during the preoperative period may provide additional information for surgical planning and assist in identifying patients who truly require trochleoplasty, avoiding unnecessary procedures and reducing morbidity and the risk of surgical complications.

Ex vivo assessment of trochlear groove depth has been used in dogs as the gold standard for comparison with imaging methods, such as ultrasonography and radiography [22,25]. The authors consider ex vivo measurement a reliable reference method for this type of comparison, as the evaluator directly visualizes the anatomy and the exact measurement point. Therefore, the use of ex vivo measurements as a reference method is essential for validating the accuracy of different imaging techniques and determining which method shows the highest agreement with the true anatomical structure.

Ultrasonography is an operator-dependent imaging modality in which the examiner’s experience may influence image acquisition [16], measurement performance, and, consequently, the reproducibility of results in clinical practice. However, ultrasonography is a non-invasive imaging method free of ionizing radiation that, in most cases, can be performed without the need for sedation, providing greater patient safety [30,31,32,33]. In addition to this advantage, the technique allows evaluation of articular cartilage [33,34,35], a characteristic that may have contributed to the similarity observed between ultrasonographic measurements at 90° and ex vivo evaluation with the stifle positioned at 90°. In contrast, no similarity was observed between measurements obtained with the stifle positioned at 45°. A possible explanation for this finding is that, at this angle, the position of the patella in relation to the transducer may hinder adequate delimitation of the trochlear groove due to partial superimposition of the patella. Furthermore, small variations in transducer angulation, required to minimize the posterior acoustic shadowing artifact produced by the patella over the trochlear groove, may influence the measurements, contributing to the discrepancy observed in relation to ex vivo evaluation at 45°.

The skyline radiographic projection of the stifle is used for the assessment of trochlear groove depth in dogs [25,35,36]; however, the authors are unaware of studies evaluating the applicability of this projection in cats. In the present study, measurements obtained by skyline radiography showed similarity with ex vivo evaluation performed with the stifle positioned at 90° of flexion. This result suggests that radiography may represent a useful alternative for the preoperative assessment of trochlear groove depth in felines, particularly when ultrasonography is unavailable. The absence of a statistically significant difference (*p* = 0.157) was likely related to the similarity between the angulation of the radiographic beam in the skyline projection and the joint positioning adopted during ex vivo evaluation at 90° of flexion, favoring a more similar localization of the trochlear groove assessment area. In contrast, this similarity was not observed in relation to ex vivo evaluation performed with the stifle positioned at 45° of flexion, suggesting that the degree of joint flexion may influence measurements of trochlear groove depth.

When comparing computed tomography measurements with those obtained from ex vivo evaluation, differences between the means were observed, with higher values obtained by computed tomography at both 45° and 90° of stifle flexion. A possible explanation for this finding is the use of a CT scanner with a lower number of channels and greater slice thickness, factors that reduce spatial resolution and image definition [37], potentially compromising the accurate identification of the anatomical landmarks used for measuring trochlear groove depth. In addition, the correspondence between the measurement plane obtained by computed tomography and that used during ex vivo evaluation may not have been exactly the same, making direct comparison between methods more challenging. Therefore, computed tomography measurements should be interpreted with caution during surgical planning, as small differences may influence the decision regarding the need for trochleoplasty. The authors believe that the use of multidetector computed tomography systems with thinner slice acquisition and isotropic voxel reconstruction may improve the accuracy of morphometric measurements compared with the equipment used in the present study.

The discrepancy observed between the measurements obtained by computed tomography and the ex vivo evaluations may be related not only to the technical parameters of the equipment but also to the methodology used for measurement. Although multiplanar reconstruction allowed the acquisition of images in the axial plane and identification of the deepest point of the trochlear groove, differences in the orientation of the reconstructed plane or in the selected proximodistal level may have influenced the measurements obtained. The use of reconstructions in mutually perpendicular planes and three-dimensional reconstructions could help to more accurately identify the region of greatest trochlear depth and standardize the measurements. Therefore, the CT results should be interpreted with caution, as small differences in trochlear groove depth measurements may have implications for surgical planning and the decision regarding the need for trochleoplasty.

This study has some limitations that should be considered. First, the sample size was relatively small, which may limit the generalizability of the results and the establishment of definitive reference values for trochlear groove depth assessment in cats. Additionally, intra- and interobserver repeatability analyses were not performed, preventing the evaluation of the reproducibility and reliability of the measurements obtained using the different imaging techniques. Furthermore, only a single slice thickness was used for computed tomography acquisition. Variations in computed tomography acquisition parameters, particularly slice thickness, may influence spatial resolution and image quality, potentially affecting the accuracy of trochlear groove depth measurements. Future studies involving cats with naturally occurring patellar luxation are needed to assist in the interpretation of the data obtained in this study, as well as to evaluate the impact of trochlear groove conformation and flattening on the occurrence and severity of patellar luxation in cats.

## 5. Conclusions

The results of this study indicate that the joint flexion angle has an important influence on the measurement of the trochlear groove in cats. At 45° of flexion, measurements obtained by ultrasonography, radiography, and computed tomography differed significantly from the reference measurements, whereas at 90° of flexion, ultrasonographic and radiographic measurements showed greater agreement with the reference measurements. Therefore, standardization of joint positioning and flexion angle should be considered when evaluating the trochlear groove in cats. Further studies with larger sample sizes and assessment of intra- and interobserver reliability are warranted to determine the reproducibility and clinical applicability of these imaging methods.

## Figures and Tables

**Table 1:** Comparison of trochlear groove depth measurements obtained by radiography, ultrasonography, and computed tomography with macroscopic evaluation in cats (cm).

	Mean ± SD	Ex Vivo Measurement	*p* < 0.05
X-ray vs. Macro 45°	0.159 ± 0.024	0.094 ± 0.012	<0.001
X-ray vs. Macro 90°	0.159 ± 0.024	0.150 ± 0.019	0.157
US 45° vs. Macro 45°	0.104 ± 0.015	0.094 ± 0.012	0.005
US 90° vs. Macro 90°	0.151 ± 0.020	0.150 ± 0.019	0.832
CT vs. Macro 45°	0.163 ± 0.015	0.094 ± 0.012	<0.001
CT vs. Macro 90°	0.163 ± 0.015	0.150 ± 0.019	0.009

SD: standard deviation; X-ray: radiography; US: ultrasonography; CT: computed tomography; and Macro: macroscopic.

**Table 2:** Comparative evaluation of femoral trochlear groove depth between the right and left pelvic limbs of cats, measured by radiography, ultrasonography (45° and 90°), computed tomography, and macroscopic evaluation (cm).

	Mean ± SD Right	Mean ± SD Left	*p* < 0.05
X-ray	0.164 ± 0.028	0.156 ± 0.019	0.402
US 45°	0.102 ± 0.013	0.109 ± 0.016	0.165
US 90°	0.152 ± 0.021	0.149 ± 0.020	0.558
CT	0.162 ± 0.015	0.163 ± 0.017	0.917
Macro 45°	0.092 ± 0.12	0.095 ± 0.13	0.240
Macro 90°	0.153 ± 0.020	0.146 ± 0.018	0.221

SD: standard deviation; X-ray: radiography; US: ultrasonography; CT: computed tomography; and Macro: macroscopic.

**Table 3:** Comparison among imaging modalities for the evaluation of the trochlear groove in cats.

Imaging Modalities		*p* < 0.05
	US 45°	<0.001
X-ray	US 90°	0.667
	CT	0.999
US 45°	US 90°	<0.001
	CT	<0.001
US 90°	CT	0.077

X-ray: radiography; US: ultrasonography; and CT: computed tomography.

## Data Availability

The raw data supporting the conclusions of this article will be made available by the authors upon request.
